# Diabetes and pre-diabetes among adults reaching health centers in Luanda, Angola: prevalence and associated factors

**DOI:** 10.1038/s41598-020-61419-y

**Published:** 2020-03-12

**Authors:** Claudia Robbiati, Giovanni Putoto, Natália Da Conceição, António Armando, Giulia Segafredo, Andrea Atzori, Francesco Cavallin

**Affiliations:** 1 Doctors with Africa, Luanda, Angola; 2 Doctors with Africa, Padova, Italy; 3 National Directory of Public Health, Ministry of Health of Angola, Luanda, Angola; 4 Independent statistician, Solagna, Italy

**Keywords:** Type 2 diabetes, Epidemiology

## Abstract

With the lack of surveys, surveillance program and/or statistical data, epidemiologic studies can provide a better understanding of diabetes in Sub-Saharan Africa. This was a cross-sectional survey to determine prevalence of diabetes and impaired fasting glucose (IFG) among adults attending six health centres in six different districts of Luanda (Angola) during August-November 2018, followed by a case-control study to assess the risk factors for IFG and diabetes in a subgroup of subjects not receiving treatment for diabetes. Factors associated with diabetes/IFG were assessed using a generalized ordered logit model and the effects were expressed as odds ratios (OR_1_ for IFG/diabetes vs. no IFG/diabetes; OR_2_ for diabetes vs. no diabetes) with 95% CI (confidence interval). Some 1,803 participants were included in the survey. Prevalence of diabetes was 12.0% (95%CI 10.5% to 13.5%) and prevalence of IFG was 9.0% (95%CI 7.7% to 10.4%). Older age (OR_1_ = OR_2_ 1.03, 95%CI 1.02 to 1.04), higher weight (OR_1_ = OR_2_ 1.01, 95%CI 1.01 to 1.03), having measured glycaemia before (OR_2_ 2.07, 95%CI 1.29 to 3.31), feeling polyuria (OR_1_ 1.93, 95%CI 1.13 to 3.28; OR_2_ 2.18, 95%CI 1.32 to 3.59), feeling polydipsia (OR_1_ 1.92, 95%CI 1.16 to 3.18), feeling weakness (OR_1_ = OR_2_ 2.22, 95%CI 1.39 to 3.55), consumption of free-sugars food/beverages (OR_1_ = OR_2_ 2.34, 95%CI 1.44 to 3.81) and time spent seated (OR_1_ 1.80, 95%CI 1.17 to 2.76) were associated with increased likelihood of diabetes and/or IFG, while eating vegetables was associated with decreased likelihood of IFG or diabetes (OR_1_ = OR_2_ 0.69, 95%CI 0.47 to 0.99). In conclusion, the high prevalence of diabetes and IFG, with common unawareness of the disease, calls for appropriate interventions in Angolan urban settings. Further research may evaluate the impact of context-specific factors to enhance intervention strategies and feed the results into local health policies. In addition, such information may be useful for selecting high-risk subjects to test.

## Introduction

Diabetes is among the non-communicable diseases (NCDs) with an increasing prevalence in Sub-Saharan Africa (SSA)^1^. Some 15.5 million adults aged 20–79 years were estimated to be living with diabetes in the Africa Region in 2017^2^, and this number is expected to rise to 23.9 million by 2030^3^. However, these figures have a high degree of uncertainty since 66.7% of people with diabetes in Africa are not diagnosed with the disease^4^. This is mostly due to inadequate NCDs screening programs and awareness in many African countries^5^. Epidemiological evidence about diabetes and other NCDs in SSA is scant, despite their increasing burden and the need for reliable data to develop policies, guidelines and interventions^6^.

The World Health Organisation (WHO) estimates a diabetes prevalence of 5.6% in Angola^7^, but this figure is derived by extrapolation due to the lack of epidemiological data. Angola, as other SSA countries, is experiencing an epidemiological transition and a double burden of communicable and non-communicable diseases especially in urban settings, due to changes in lifestyle, diet and physical activity^6^. The diabetes growing challenge undermines the already weak Angolan health system. The lack of national policies and guidelines on diabetes, on top of the absence of accurate estimates of disease prevalence^7^, highlights the urgency to develop proper programs for the early detection of diabetes and impaired fasting glucose (IFG) (or pre-diabetes). IFG is a condition that precedes diabetes and increases the risk to develop it, therefore identifying people with IFG and associated risk factors is a preliminary step to define prevention programs to stop the progression of diabetes^8^.

With the lack of surveys, surveillance program and/or statistical data, epidemiologic studies (such as cross-sectional and longitudinal) can provide a better understanding of diabetes in Angola. This study aimed to investigate the prevalence of IFG and diabetes in people attending healthcare facilities in Luanda, the capital city of Angola. Factors associated with IFG and diabetes were also investigated among people not receiving treatment for diabetes.

## Methods

### Study design

This was a cross-sectional survey to determine prevalence of diabetes and IFG among people attending six health centres in six different districts of Luanda (Angola), followed by a case-control study to assess the factors associated with IFG and diabetes in a subgroup of subjects not receiving treatment for diabetes.

### Setting

The study was conducted in the urban area of Luanda (Angola), with a population of approximately 7 million people. According to national authorities, the capital city of Angola has the highest diabetes mortality rate in the country (3.4%)^9^. One health center was randomly chosen in each of the six districts. People attending the six health centres between August 2018 and November 2018 were evaluated for inclusion in the study.

### Participants

The study population included adult men and women older than 18 years attending the six health centres between August 2018 and November 2018. Pregnant women and people not fasting for at least 8 hours were excluded from the study.

### Cross-sectional survey

All participants were included in the cross-sectional survey to determine the prevalence of IFG and diabetes. Fasting blood glucose (FBG) levels were measured by professional nurses using a glucometer (Infopia, South Korea). Diabetes measurements and definitions were based on WHO guidelines^10^. IFG was defined as FBG levels between 110–125 mg/dl. Diabetes was defined as FBG levels ≥126 mg/dl or a self-report of previous diagnosis of diabetes by a health care professional or currently receiving treatment for diabetes.

### Case-control study

A subgroup of participants was selected for the investigation of factors associated with IFG and diabetes among those who were not receiving treatment for diabetes, as follows. All subjects with IFG were included. The chosen ratio of participants with IFG, diabetes and without IFG /diabetes was 1:1:1. For each participant with IFG, the next participant without IFG /diabetes was also included in the second step. Participants with diabetes were included in the second step and “linked” with the closest (past or next) participant with IFG, to warrant the achievement of the set 1:1:1 ratio. This approach was chosen to overcome the logistics issues of evaluating the factors in all participants of the cross-sectional survey.

A set of information of interest for IFG/diabetes (demographics, clinical parameters, clinical information, diet habits and physical activity) was collected by professional nurses using a case-report form. Demographics included age, sex, marital status, employment, education, number of children and number of people living at home. Clinical parameters included weight, body mass index (BMI), waist circumference, systolic blood pressure (SBP), diastolic blood pressure (DBP) and heart rate. Clinical information included having already heard about diabetes, having glycaemia measured before, losing weight, and feeling polyuria, polydipsia and weakness. Diet habits included eating vegetables, fruits, free-sugars food, adding refined salt in meals and drinking alcohol or free-sugars beverages daily. Tobacco use was self-reported. Information on physical activity included whether the subject usually performed physical activity for at least 30 minutes per day, and how long the subject remained seated daily (<2 hours, 2–5 hours,>5 hours). Demographics, clinical information, diet habits and physical activity were self-reported, while clinical parameters were measured by a professional nurse. All data were collected by a professional nurse before participants went into the doctor’s consultation room. Data collection was coordinated and supervised by one research assistant.

### Sample size

The sample size was calculated according to the primary aim of reporting the prevalence of diabetes and IFG in the cross sectional survey. Assuming a proportion of participants with IFG between 60 and 90 per 1,000 subjects, some 963 to 1,400 participants needed to be enrolled to estimate a 95% confidence interval (CI) not wider than 30 per 1,000 subjects. Assuming a proportion of participants with diabetes between 80 and 120 per 1,000 subjects, some 1,257 to 1,803 participants needed to be enrolled to estimate a 95% CI not wider than 30 per 1,000 subjects. The largest of the calculated sample sizes (1,803 participants) was chosen.

### Statistical analysis

In the cross sectional survey, the proportions (with 95% confidence interval, CI) of participants with IFG, diabetes or without IFG/diabetes were estimated in all sample.

In the case-control study, factors associated with IFG and diabetes were investigated in the subgroup of participants, who were divided in three study groups (no IFG/diabetes; IFG; diabetes). Continuous data were reported as median and interquartile range (IQR), while categorical data as number and percentage. Continuous data were compared among study groups using Kruskal-Wallis test, and categorical data using Chi-square test or Fisher’s exact test, as appropriate.

Multivariable regression was performed to identify independent predictors of study group (no IFG/diabetes; IFG; diabetes) among a set of clinically relevant variables. These included age, sex, marital status, employment, weight, BMI, waist circumference, SBP, DBP, having glycaemia measured before, polyuria, weight loss, polydipsia, weakness, eating vegetables, eating fruits, consuming free-sugars food and beverages, alcohol assumption, adding refined salt in meals, doing physical activities for at least 30 minutes and time spent seated. Some of them (DBP, BMI, waist circumference, marital status, employment) were not included in the initial model due to collinearity with SBP, weight or age. Eating free-sugars food and drinking free-sugars beverages conveyed the same meaning, thus were combined in a single variable (named “consuming free-sugars food/beverages”). As proxy for sedentary habits, time spent seated was preferred to “doing physical activities for at least 30 minutes”. Model selection was performed by AIC reduction. Owing to the ranked outcome, the ordered logistic regression model was initially chosen. The proportional odds assumption was checked graphically because statistical tests for this purpose have been criticized to be un-conservative^11^. The proportional odds assumption was not satisfied for some variables, thus a generalized ordered logit (partial proportional odds) model was estimated^12^. Effects sizes were reported as odds ratios (OR) with 95% confidence intervals (CI), where OR_1_ indicated the odds ratio for IFG/diabetes vs. no IFG/diabetes, and OR_2_ indicated the odds ratio for diabetes vs. no diabetes. The analysis estimated two different odds ratios (OR_1_ and OR_2_) for the explanatory variables violating the proportional odds assumption, while equal odd ratios (OR_1_ = OR_2_) were reported for all other explanatory variables not violating the assumption. Missing data were not imputed. All test were 2-sided and a p-value less than 0.05 was considered statistically significant. Statistical analysis was performed using R 3.5 (R Foundation for Statistical Computing, Vienna, Austria)^13^.

### Ethics

This study was approved by the National Public Health Directorate of the Ministry of Health of Angola and by the Ethics Committee of the Ministry of Health of Angola (number 21/2018). Each participant signed a full informed consent form. All methods were performed in accordance with the relevant guidelines and regulations. The study used anonymized data and no identifiable data were collected.

## Results

### Cross sectional survey

A total of 1,803 participants were included in the cross sectional survey and had their FBG levels tested between August 2018 and November 2018. Diabetes was diagnosed in 216 participants (prevalence 12.0%, 95% CI 10.5% to 13.5%) and IFG in 162 participants (prevalence 9.0%, 95% CI 7.7% to 10.4%), while 1,425 participants had no IFG/diabetes (prevalence 79.0%, 95% CI 77.1% to 80.9%) (Table 1).

**Table 1:** Prevalence of IFG and diabetes among 1,803 participants who attended the six health centers in Luanda (Angola) during August-November 2018 and had their FBG levels tested (cross sectional survey).

	N of participants	Estimated prevalence	95% confidence interval
No IFG/diabetes	1,425	79.0%	77.1% to 80.9%
IFG	162	9.0%	7.7% to 10.4%
Diabetes	216	12.0%	10.5% to 13.5%

Among the 216 participants with diabetes: 144 (66.7%) had FBG ≥ 126 mg/dl without previous diagnosis or treatment for diabetes; 27 (12.5%) were not receiving treatment for a previously diagnosed diabetes and had FBG ≥ 126 mg/dl; 27 (12.5%) had FBG ≥ 126 mg/dl despite receiving treatment for diabetes; 18 (8.3%) were receiving treatment for diabetes and had FBG ≤ 125 mg/dl.

### Case-control study

Factors associated with IFG and diabetes were investigated in a subgroup of 486 participants (162 per study group) as described in Methods. Participant characteristics are reported in Table 1. Age (p < 0.0001), marital status (p = 0.001) and employment (p < 0.0001) were different among the study groups (Table 2).

**Table 2:** Characteristics of 486 participants not receiving treatment for diabetes who attended the six health centers in Luanda (Angola) during August-November 2018 (case-control study).

	No IFG/diabetes	IFG	Diabetes	p-value
N	162	162	162	—
Age, years^a,b^	33 (26–48)	42 (30–55)	46 (35–58)	<0.0001
Male:female	40:122	42:120	50:112	0.42
Marital status^c^				0.001
Single	136 (84)	121 (76)	100 (64)	
Married	20 (12)	29 (18)	39 (25)	
Widow/divorced	6 (4)	9 (6)	17 (11)	
Employment^d^				<0.0001
Student	26 (16)	14 (9)	11 (7)	
Worker	52 (33)	66 (43)	60 (39)	
Housewife/other	68 (43)	36 (23)	44 (29)	
Retired/unemployed	13 (8)	38 (25)	38 (25)	
Education^e^				0.10
None	6 (4)	24 (16)	17 (11)	
Primary	35 (24)	25 (16)	30 (20)	
Secondary 1^st^ cicle	37 (26)	35 (24)	40 (27)	
Secondary 2^nd^ cicle	46 (32)	49 (33)	44 (30)	
University	21 (14)	16 (11)	18 (12)	
Number of children^f^				0.06
0	28 (18)	20 (13)	15 (10)	
1	11 (7)	17 (11)	12 (8)	
2	24 (15)	14 (9)	12 (8)	
≥3	94 (60)	105 (67)	112 (74)	
Number of people living at home^g^				0.53
1–3	25 (16)	23 (16)	31 (20)	
4–6	76 (48)	65 (42)	65 (42)	
≥7	56 (36)	65 (42)	59 (38)	

Data expressed as n(%) or ^a^ median (IQR). Data not available in ^b^5, ^c^9, ^d^20, ^e^43, ^f^22 and ^g^21 participants. The comparisons were performed using Kruskal-Wallis test, Chi-square test or Fisher’s exact test, as appropriate.

Weight (p < 0.0001), BMI (p = 0.01), waist circumference (p = 0.01), systolic blood pressure (p = 0.0002), diastolic blood pressure (p = 0.0003) and heart rate (p < 0.0001) were different among the study groups, with the increasing values in participants with IFG and diabetes with respect to those with no IFG/diabetes (Table 3).

**Table 3:** Clinical parameters of 486 participants not receiving treatment for diabetes who attended the six health centers in Luanda (Angola) during August-November 2018 (case-control study).

	No IFG/diabetes	IFG	Diabetes	p-value
N	162	162	162	—
Weight, kg^a^	60 (54–69)	68 (56–81)	68 (59–78)	<0.0001
BMI, kg/m^2b^	22.4 (19.4–25.8)	24.1 (20.6–28.0)	23.8 (20.9–27.4)	0.01
Waist circumference, cm ^c^	77 (63–89)	79 (60–95)	84 (69–98)	0.01
Systolic blood pressure, mmHg^a^	120 (110–136)	130 (113–147)	130 (114–150)	0.0002
Diastolic blood pressure, mmHg^d^	73 (63–80)	78 (70–90)	80 (70–90)	0.0003
Heart rate, bpm^e^	70 (62–79)	75 (65–82)	80 (67–92)	<0.0001

Data expressed as median (IQR). Data not available in ^a^10, ^b^42, ^c^38, ^d^12 and ^e^26 participants. The comparisons were performed using Kruskal-Wallis test.

**Figure 1:**
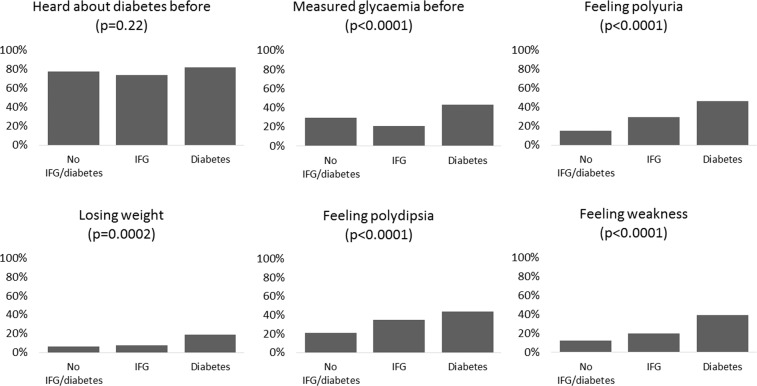
Clinical information of 486 participants not receiving treatment for diabetes who attended the six health centers in Luanda (Angola) during August-November 2018 (case-control study).

The proportion of participants who already heard about diabetes was not different in study groups (p = 0.22), but having glycaemia measured before (p < 0.0001) was more frequent in participants with diabetes (Fig. 1). The occurrence of symptoms (i.e. polyuria, weight loss, polydipsia and weakness) was different among study groups, with higher rates in subjects with IFG or diabetes (Fig. 1). Only 14 participants were smokers. All numeric data are reported in Supplementary Information.

**Figure 2:**
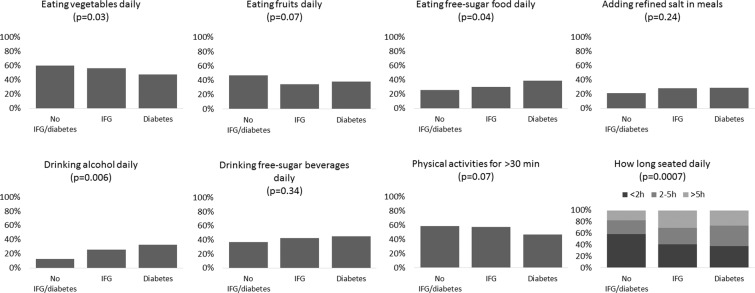
Diet habits and physical activity of 486 participants not receiving treatment for diabetes who attended the six health centers in Luanda (Angola) during August-November 2018 (case-control study).

Eating vegetables (p = 0.03), eating free-sugars food (p = 0.04), alcohol assumption (p = 0.006) and time spent seated (p = 0.0007) were different among study groups (Fig. 2). Eating vegetables was more frequent in participants without IFG/diabetes, who also reported shorter time spent seated and less frequent assumption of sweetened products and alcohol (Fig. 2). Eating fruit (p = 0.07) and doing physical activities for at least 30 minutes (p = 0.07) showed small (non-statistically significant) differences among study groups (Fig. 2). There were no statistically significant differences in drinking free-sugars beverages (p = 0.34) and adding refined salt in meals (p = 0.24) among study groups (Fig. 2). All numeric data are reported in Supplementary Information.

Multivariable analysis was performed to identify predictors of study group (no IFG/diabetes; IFG; diabetes) among a set of clinically relevant variables (as described in Methods). Sex, SBP, weight loss, eating fruits and adding refined salt in meals were excluded to the model due to AIC reduction. The final model is shown in Table 4. Older age (OR_1_ = OR_2_ 1.03, 95% CI 1.02 to 1.04) and higher weight (OR_1_ = OR_2_ 1.01, 95% CI 1.01 to 1.03) were associated with increased likelihood of IFG or diabetes. Having measured glycaemia before was associated with increased risk of diabetes (OR_2_ 2.07, 95% CI 1.29 to 3.31) but no significant difference was found for IFG/diabetes vs. no IFG/diabetes (OR_1_ 0.75, 95% CI 0.47 to 1.20). Feeling polyuria was associated with increased likelihood of IFG/diabetes (OR_1_ 1.93, 95% CI 1.13 to 3.28) and of diabetes (OR_2_ 2.18, 95% CI 1.32 to 3.59). Feeling polydipsia was associated with increased likelihood of IFG/diabetes (OR_1_ 1.92, 95% CI 1.16 to 3.18) but no significant difference was found for diabetes vs. no diabetes (OR_2_ 1.43, 95% CI 0.87 to 2.36). Feeling weakness was associated with increased likelihood of IFG or diabetes (OR_1_ = OR_2_ 2.22, 95% CI 1.39 to 3.55). Eating vegetables daily was associated with decreased likelihood of IFG or diabetes (OR_1_ = OR_2_ 0.69, 95% CI 0.47 to 0.99), while daily consumption of free-sugars food/beverages was associated with increased likelihood of IFG or diabetes (OR_1_ = OR_2_ 2.34, 95% CI 1.44 to 3.81). Remaining seated for more than 2 hours per day was associated with increased likelihood IFG/diabetes (OR_1_ 1.80, 95% CI 1.17 to 2.76) but no significant difference was found for diabetes vs. no diabetes (OR_2_ 0.95, 95% CI 0.61 to 1.50).

**Table 4:** Multivariable analysis of risk factors of IFG and diabetes among 486 participants not receiving treatment for diabetes who attended the six health centers in Luanda (Angola) during August-November 2018 (case-control study).

	Risk factors for IFG/diabetes		Risk factors for diabetes	
	OR_1_ (95% CI)	p-value	OR_2_ (95% CI)	p-value
Age, years	1.03 (1.02 to 1.04)	<0.0001	1.03 (1.02 to 1.04)	<0.0001
Weight, kg	1.01 (1.01 to 1.03)	<0.0001	1.01 (1.01 to 1.03)	<0.0001
Measured glycaemia before:		0.23		0.003
No	Reference		Reference	
Yes	0.75 (0.47 to 1.20)		2.07 (1.29 to 3.31)	
Feeling polyuria:		0.01		0.002
No	Reference		Reference	
Yes	1.93 (1.13 to 3.28)		2.18 (1.32 to 3.59)	
Feeling polydipsia:		0.01		0.16
No	Reference		Reference	
Yes	1.92 (1.16 to 3.18)		1.43 (0.87 to 2.36)	
Feeling weakness:		0.0008		0.0008
No	Reference		Reference	
Yes	2.22 (1.39 to 3.55)		2.22 (1.39 to 3.55)	
Eating vegetables daily:		0.04		0.04
No	Reference		Reference	
Yes	0.69 (0.47 to 0.99)		0.69 (0.47 to 0.99)	
Consuming free-sugars		0.0006		0.0006
food/beverages daily:	Reference		Reference	
No	2.34 (1.44 to		2.34 (1.44 to	
Yes	3.81)		3.81)	
Drinking alcohol daily:		0.14		0.14
No	Reference		Reference	
Yes	1.43 (0.89 to 2.30)		1.43 (0.89 to 2.30)	
Seated >2 hours daily:		0.007		0.84
No	Reference		Reference	
Yes	1.80 (1.17 to 2.76)		0.95 (0.61 to 1.50)	

Results from the generalized ordered logit (partial proportional odds) model. Effects sizes are reported as odds ratio (OR) with 95% confidence interval (CI). OR_1_ indicated the odds ratio for IFG/diabetes vs. no IFG/diabetes, while OR_2_ indicated the odds ratio for diabetes vs. no diabetes. The analysis estimated two different odds ratios (OR_1_ and OR_2_) for the explanatory variables violating the proportional odds assumption, while equal odd ratios (OR_1_ = OR_2_) were reported for all other explanatory variables not violating the assumption.

## Discussion

Our study revealed a high prevalence of diabetes and IFG among adults attending healthcare facilities in the capital city of Angola. Every 100 of them, diabetes was likely to be found in 12 and IFG in nine. Of note, two out of three adults with diabetes were unaware of their condition. Multivariable analysis indicated some factors associated with diabetes and/or IFG. Some were diet/lifestyle factors such as not eating vegetables daily, consumption of free-sugars food/beverages and time spent seated. Some were demographics/biometrics (i.e. older age, higher weight) or symptoms (i.e. polyuria, polydipsia, weakness), that could be used to identify adults to test. Interestingly, a previous measurement of glycaemia was associated with increased likelihood of diabetes.

Our findings contributes to the investigation of diabetes in SSA, where available figures are based on extrapolation for 32 out of 49 countries^2^. In Angola, WHO reports no policies or action plans for diabetes, and the absence of registries, surveys or surveillance on the disease^7^. In this context, epidemiologic studies can shed some light on magnitude and features of the situation. So far, four cross-sectional studies have provided some data on diabetes in Angola. High prevalence (9.2%) and low awareness of diabetes were found in adults living in Dande municipality^14^, while low prevalence (2.8%) but no awareness of diabetes were reported in a rural community^15^. Low but not-negligible prevalence of diabetes was found among healthy workers of a private tertiary health center (2.69%) and a university (5.7%)^16,17^. The present study adds on available information from rural areas or restricted to very specific subjects (i.e. workers of a health center and workers of a university) by presenting data from an urban setting. Our findings indicate a high prevalence of diabetes and IFG among adults attending health centers in the capital city of Angola, while confirming the common unawareness of the disease^4^.

Our study focused the investigation on an urban setting because many SSA counties are experiencing an increase in NCDs due to changes in diet and lifestyle associated with urbanization^6^. In the African region, the prevalence of diabetes has been found higher in urban compared to rural settings^18^, with urbanization boosting the increasing trend but also representing an opportunity to develop urban strategies conducive to healthy behaviours^6^. WHO underlines the role of regular physical activity, weight control and healthy diet in reducing the risk of diabetes^19^, but context-tailored approaches are needed to reduce the prevalence of modifiable risk factors in SSA population^20^. Within its limitations, our findings provided suggestions for identification of subjects to test and for diabetes reduction strategies in Angolan urban settings. While there is little evidence of benefit of diabetes screening in low-resource settings^5^, it is still plausible that early detection of diabetes in Luanda may improve patient’s outcomes by avoiding serious complications before diagnosis. On the other hand, there is no evidence that counselling people with IFG changes their future risk of diabetes^10^.

The present study has some limitations that should be considered. First, it is a cross-sectional study including adults attending health facilities in Luanda, thus the generalization of the findings may be limited to similar settings (i.e. adults attending health facilities in urban areas in sub-Saharan countries). The demographics of our sample might be compared with those of the population served by the health centers, in order to generalize the findings to the larger community and to identify gaps in terms of who was seeking care at the health facilities. Unfortunately, such information was not available at the time of the analysis, but further investigations may contribute to fill this gap. Second, information about the reason for attending the health facilities was not collected because the study aimed to evaluate the magnitude of IFG/diabetes burden among people attending the health facilities irrespective of the reason, in order to advise health care stakeholders. Future investigations may disclose the reasons for seeking care at the health centers in Luanda, as well as possible associations with IFG and diabetes. Third, we relied only on FBG to classify prediabetes and diabetes, while a 2-h oral glucose tolerance test (OGTT) was not conducted. Since estimated diabetes prevalence may be higher with 2-h OGTT thresholds compared with FBG thresholds^21^, our results may be considered a conservative estimate of the diabetes prevalence among adults attending the health centers in Luanda. Forth, the cross-sectional nature precludes any causal association, and the reliance of self-reported information on diet and physical activity may bias some results in the analysis of risk factors.

Nevertheless, the high prevalence of diabetes and IFG - coupled with large unawareness - in the capital city of Angola calls for improvements in prevention and identification of the disease. While population-wide screening should be avoided in low-resource settings^5^, the identification of factors associated with diabetes/IFG may help in selecting subjects to test in sub-Saharan urban settings. In addition, participants who reported a previous measurement of glycaemia resulted at increased risk of diabetes, thus confirming poor management of the disease and unmet need for diabetes care in low-resource settings^22^. With diabetes increasing in SSA, improving the understanding of the disease in the local context is essential for the implementation of proven interventions^6,19^. Moreover, risk-based interventions may be especially effective where diagnostic tools are lacking^23^. Detailed diet and lifestyle investigation with appropriate tools may provide useful information for context-tailored interventions^20,24^. SSA countries share some limitations to prevention and management options for diabetes, including poor understanding of the disease, poor control of glycaemia and other risk factors, and barriers to treatment or transport to treatment facilities^5^. Although there are examples of successful strategies in high-resource settings, further research should focus on assess whether these strategies are transferable to SSA^5,20,25,26^.

## Conclusions

The high prevalence of diabetes and IFG, with common unawareness of the disease, calls for appropriate interventions in Angolan urban settings. Further research may evaluate the impact of context-specific factors to enhance intervention strategies and feed the results into local health policies. In addition, such information may be useful for selecting high-risk subjects to test.

## Supplementary information

Supplementary information.

## Data Availability

The datasets used and/or analyzed during the current study are available from the corresponding author on reasonable request.
