# Supplementary material for: Diabetes and pre-diabetes among adults reaching health centers in Luanda, Angola: prevalence and associated factors
Source: Sci Rep. 2020 Mar 12;10:4565. doi: 10.1038/s41598-020-61419-y (PMC7067759; doi:10.1038/s41598-020-61419-y)
Supplement: Supplementary file 1 — Supplementary information. [file 41598_2020_61419_MOESM1_ESM.pdf]

SUPPLEMENTARY INFORMATION

TITLE: Diabetes and pre-diabetes among adults reaching health centers in Luanda, Angola: prevalence and associated factors.

Authors: Claudia Robbiati 1, Giovanni Putoto 2, Natália da Conceição 3, António Armando 3, Giulia Segafredo 2, Andrea Atzori 2, Francesco Cavallin 4

Institutions:

1. Doctors with Africa, Luanda, Angola
2. Doctors with Africa, Padova, Italy
3. National Directory of Public Health, Ministry of Health of Angola, Luanda, Angola
4. Independent statistician, Solagna, Italy

Corresponding author: Claudia Robbiati,

Doctors with Africa, Luanda, Angola

Address: Rua Projectada A3, Casa n 2, Bairro Morro Bento II, Luanda (Angola)

Email: c.robbiati@cuamm.org

Supplementary Table 1. Clinical information of 486 participants not receiving treatment for diabetes who attended the six health centers in Luanda (Angola) during August-November 2018 (case-control study). These data refer to Figure 1.

	No IFG/diabetes	IFG	Diabetes	p-value
N	162	162	162	-
Has he/she heard about diabetes before?				0.22
No	36	42	29	
Yes	126	120	133	
Has he/she measured glycaemia before?				<0.0001
No	114	128	92	
Yes	48	34	70	
Does he/she feel polyuria?				<0.0001
No	137	114	87	
Yes	25	48	75	
Is he/she losing weight?				0.0002
No	152	150	131	
Yes	10	12	31	
Does he/she feel polydipsia?				<0.0001
no	128	105	91	
Yes	34	57	71	
Does he/she feel weakness?				<0.0001
No	142	130	98	
Yes	20	32	64	

Data expressed as n participants.

Supplementary Table 2. Diet and physical activity of 486 participants not receiving treatment for diabetes who attended the six health centers in Luanda (Angola) during August-November 2018 (case-control study). These data refer to Figure 2.

	No IFG/diabetes	IFG	Diabetes	p-value
N	162	162	162	-
Does he/she eat vegetables daily?				0.03
No	67	71	85	
Yes	101	91	77	
Does he/she eat fruits daily?				0.07
No	86	106	100	
Yes	76	56	62	
Does he/she eat free-sugars food daily?				0.04
No	120	113	199	
Yes	42	49	63	
Does he/she add refined salt in the meals?				0.24
No	127	116	115	
Yes	35	46	47	
Does he/she drink alcohol daily?				0.006
No	141	130	118	
Yes	21	32	44	
Does he/she drink free-sugars beverages daily?				0.34
No	102	93	89	
Yes	60	69	73	

Does he/she do any physical activity for at least 30 min?				0.07
No	67	69	86	
Yes	95	93	76	
How long does he/she remain seated per day?				0.0007
Less than 2 hours	95	66	61	
Between 2-5 hours	39	46	58	
More than 5 hours	28	50	43	

Data expressed as n participants.
